# The Anticancer Potential of Doxycycline and Minocycline—A Comparative Study on Amelanotic Melanoma Cell Lines

**DOI:** 10.3390/ijms23020831

**Published:** 2022-01-13

**Authors:** Jakub Rok, Zuzanna Rzepka, Justyna Kowalska, Klaudia Banach, Artur Beberok, Dorota Wrześniok

**Affiliations:** Department of Pharmaceutical Chemistry, Faculty of Pharmaceutical Sciences in Sosnowiec, Medical University of Silesia, Jagiellońska 4, 41-200 Sosnowiec, Poland; zrzepka@sum.edu.pl (Z.R.); jkowalska@sum.edu.pl (J.K.); kbanach@sum.edu.pl (K.B.); abeberok@sum.edu.pl (A.B.); dwrzesniok@sum.edu.pl (D.W.)

**Keywords:** doxycycline, minocycline, melanoma, apoptosis, caspases, cell cycle, MITF, ERK1/2, LC3A/B

## Abstract

Malignant melanoma is still a serious medical problem. Relatively high mortality, a still-growing number of newly diagnosed cases, and insufficiently effective methods of therapy necessitate melanoma research. Tetracyclines are compounds with pleiotropic pharmacological properties. Previously published studies on melanotic melanoma cells ascertained that minocycline and doxycycline exerted an anti-melanoma effect. The purpose of the study was to assess the anti-melanoma potential and mechanisms of action of minocycline and doxycycline using A375 and C32 human amelanotic melanoma cell lines. The obtained results indicate that the tested drugs inhibited proliferation, decreased cell viability, and induced apoptosis in amelanotic melanoma cells. The treatment caused changes in the cell cycle profile and decreased the intracellular level of reduced thiols and mitochondrial membrane potential. The exposure of A375 and C32 cells to minocycline and doxycycline triggered the release of cytochrome c and activated initiator and effector caspases. The anti-melanoma effect of analyzed drugs appeared to be related to the up-regulation of ERK1/2 and MITF. Moreover, it was noticed that minocycline and doxycycline increased the level of LC3A/B, an autophagy marker, in A375 cells. In summary, the study showed the pleiotropic anti-cancer action of minocycline and doxycycline against amelanotic melanoma cells. Considering all results, it could be concluded that doxycycline was a more potent drug than minocycline.

## 1. Introduction

Malignant melanoma is a skin cancer derived from highly specialized cells called melanocytes [1]. Most melanocytes are localized in the basal layer of the epidermis. Their main function is the synthesis of the biopolymer pigment melanin, which is delivered to the surrounding keratinocytes where it protects skin cells from harmful factors [2,3,4]. The malignant transformation of melanocytes can be triggered by different environmental factors, including UV radiation, heavy metals, or pesticides. Moreover, geographical location, skin phototypes I and II, intermittent and intense sun exposure, numerous pigment nevi, genetic susceptibility, or immunosuppression also belong to risk factors for melanoma development [5,6]. The process of melanocyte transformation to melanoma cells is based on genetic and epigenetic changes. The molecular studies of melanoma pathology have contributed to the classification, diagnosis, and targeted therapy [7,8].

Despite the growing knowledge about the causes and treatment of melanoma, the epidemiological data are still unsatisfactory and distressing. Melanoma is also classified as the 19th most common cancer worldwide. Moreover, it is responsible for 65% of deaths related to skin cancer, despite the fact that it accounts for only less than 5% of diagnosed skin cancers. This makes it still the leading cause of death in skin cancer patients [9]. The continual increase in melanoma cases reflects the field of public health in the United States, Australia, New Zealand, and Europe. In the United States, where melanoma is the 5th most common cancer in males and 6th in females, the costs of the treatment were estimated to be about USD 3.3 billion [10,11]. Statistical data for the US in 2021 indicates that estimated numbers of new cases and deaths from melanoma were over 100,000 and 7000, respectively [12]. This means an increase in newly diagnosed patients with melanoma of 62% when compared with 2011 [13]. It is worth mentioning that the COVID pandemic, lockdown, and many implemented restrictions decreased the number of diagnosed and treated skin cancers, including melanoma [14].

High mortality and a relatively low survival of patients with melanoma are observed, despite the common availability of various therapies. It was found that patients with stage IIID had a melanoma-specific 5-year survival of about 32% [15]. Currently, approved melanoma therapy includes surgical excision, radiation therapy, and chemotherapy with dacarbazine, interferons, immune checkpoint therapy (e.g., ipilimumab, nivolumab, pembrolizumab), and targeted therapy (e.g., dabrafenib, vemurafenib, dabrafenib) [16,17,18]. Unfortunately, even innovative and combined therapies are associated with selective usage, limited efficacy, adverse events, and the development of resistance [19,20,21,22,23]. Thus, the search for new therapies is still ongoing, which includes, among others, cell therapy, cytokines, and drug repurposing [24,25].

Doxycycline (Doxy) and minocycline (Mino) were introduced into medicine as more potent, active, and stable semisynthetic tetracycline antibiotics [26]. The molecular mechanism of antibacterial action is related to the inhibition of mRNA translation. The antibiotics impede the binding of elongator tRNA to bacterial ribosomes and inhibit translation initiation, among other things, by their allosteric influence on the IF3 layout of the 30S subunit [27]. In general, the incidence of adverse effects caused by minocycline and doxycycline is very low [28]. Due to their good tolerance and the wide antibacterial spectrum, they have found many applications in human and veterinary medicine [29]. In addition, they show many non-antibiotic properties, including anti-inflammatory, anti-oxidant, neuroprotective, and immunomodulatory effects [30]. These activities make it possible to use the drugs in the treatment of osteoarthritis, neuropsychiatric disorders, multiple sclerosis, or COVID-19 [31,32,33,34]. Moreover, it has been reported that the non-antibiotic effects of minocycline and doxycycline also include anti-cancer activity [30,35,36]. Recently published studies and analyses considered the repurposing of minocycline and doxycycline as anti-melanoma agents [37,38,39].

The study aimed to assess the anti-melanoma potential and mechanisms of action of minocycline and doxycycline using human amelanotic melanoma cell lines.

## 2. Results

### 2.1. The Assessment of Melanoma Cell Proliferation after Exposure to Minocycline and Doxycycline

**Figure 1:**
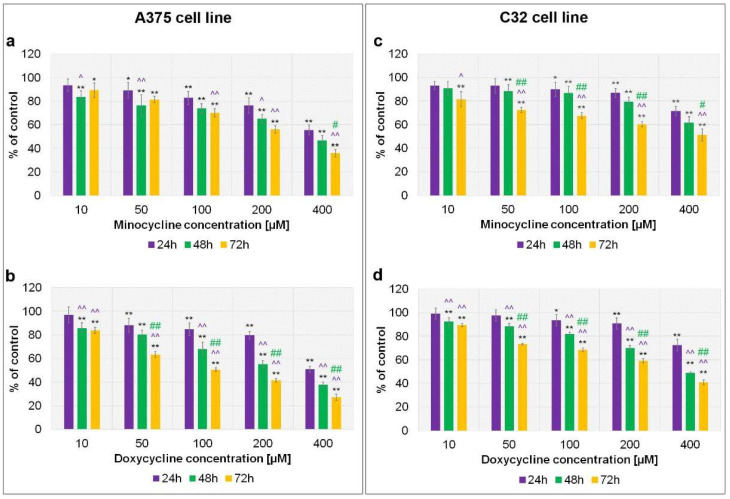
Screening analysis of melanoma cell proliferation after incubation with minocycline (**a**,**c**) and doxycycline (**b**,**d**). A375 and C32 melanoma cell were treated for 24 h, 48 h and 72 h. Mean values ± SD from three independent experiments are presented. * *p* < 0.05; ** *p* < 0.005 vs. control; ^ *p* < 0.05; ^^ *p* < 0.005 vs. 24 h; # *p* < 0.05; ## *p* < 0.005 vs. control 48 h.

A preliminary analysis of minocycline and doxycycline cytotoxicity was made using Cell Proliferation Reagent WST-1. The obtained results, presented in Figure 1, indicate that both tested drugs inhibited cell proliferation proportionally to both the concentration and the time of incubation. The effects were observed in cultures of A375 and C32 cell lines. Minocycline at the highest concentration inhibited cell proliferation to 55.6%, 46.9% and 36.0% for A375 cell line, and 71.5%, 61.1% and 51.2% for C32 cell line after 24 h, 48 h, and 72 h, respectively. Corresponding results for doxycycline were 50.9%, 37.7% and 27.1% for A375 cells, and 72.6%, 48.9% and 41.0% for C32 cells. Based on the obtained results, the values of the EC_50_ parameter were calculated for 48 h and 72 h of incubation. The calculations included logarithmic transformation and the method of best-fit values. The EC_50_ values were presented in Table 1.

The cell proliferation analysis suggested that doxycycline was more potent than minocycline. The differences were noticed especially in A375 cells after 72 h treatment. In this case, the EC_50_ parameter for doxycycline was found to be more than twice lower than for minocycline. A greater cytotoxic potential of doxycycline was also observed after 48 h and 72 h of treatment of C32 melanoma cells. It is also worth noting that the A375 cell line appeared to be more amenable to treatment with the tested tetracyclines. Both minocycline and doxycycline inhibited A375 cell proliferation more strongly than C32.

Taking into account preliminary observations, the concentrations of 200 µM and 400 µM were selected for the next experiments and analysis.

### 2.2. Analysis of Cell Cycle in A375 and C32 Melanoma Cells Incubated with Minocycline and Doxycycline

**Figure 2:**
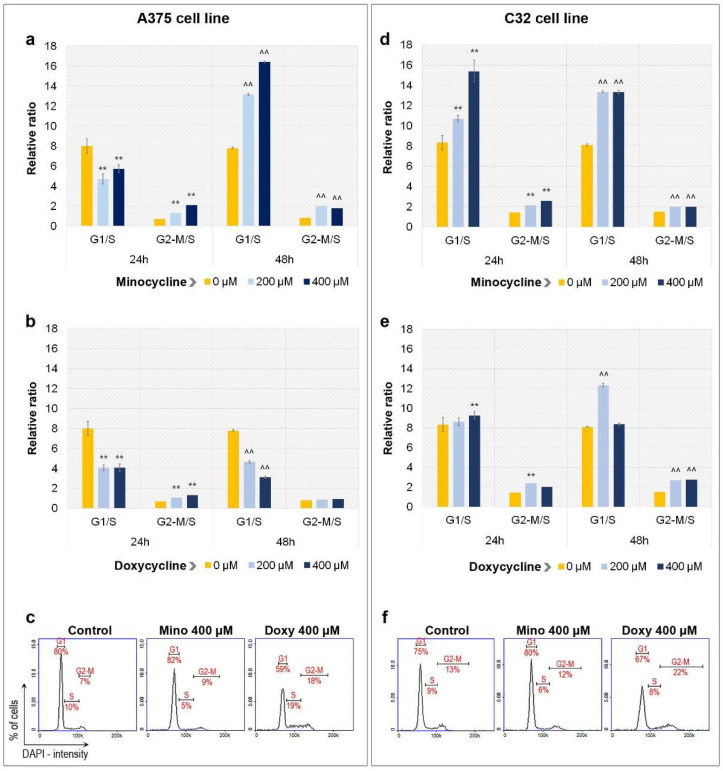
Influence of minocycline and doxycycline on cell cycle of tested amelanotic melanoma cells. The results of the analysis are presented as a relative ratio of G1/S and G2-M/S for A375 (**a**,**b**) and C32 (**d**,**e**) cell lines. Representative histograms indicate the distribution of tested cells in the individual phases of the cell cycle after 48 h treatment with the tested drugs (**c**,**f**). ** *p* < 0.005 vs. control 24 h; ^^ *p* < 0.005 vs. control 48 h.

The evaluation of the cell cycle in A375 and C32 melanoma cells was made using image cytometry. Performed analysis indicated that both minocycline and doxycycline caused changes in the cell cycle profile of tested amelanotic melanoma cell lines (Figure 2). However, the influence appeared to be different depending on the drug and the type of cells. It was observed that minocycline tended to increase the relative ratio of G1/S. The effect was noticed in A375 cells after 48 h of treatment as well as in C32 cells. The highest increase in G1/S ratio, by more than twice, was in A375 cells after 48 h of treatment with 400 µM of minocycline (16.4 vs. 7.8 in control). The same concentration of minocycline caused the highest increase in G1/S ratio in C32 melanoma cells after 24 h of treatment (15.4 vs. 8.3 in control). An exception to this was the result of the analysis of A375 cells after 24 h of treatment, in which both drug concentrations decreased the value of the ratio (4.7 and 5.7 for 200 µM and 400 µM of minocycline). It is worth noting that in all analyzed cases, minocycline increased the value of the G2-M/S ratio. The biggest difference from the control was observed for A375 cells after 24 h of treatment with 400 µM of minocycline (2.1 vs. 0.7 in control).

The effect of doxycycline on the cell cycle profile was more variable. The drug in both tested concentrations significantly decreased the value of the G1/S ratio in A375 cells. The lowest value was observed after 48 h of treatment with 400 µM (3.1 vs. 7.8 in control). In the case of the C32 cell line, an increase in G1/S ratio was found after 24 h of treatment with 400 µM of doxycycline (9.3 vs. 8.3 in control) as well as after 48 h of treatment with 200 µM of doxycycline (12.3 vs. 8.1 in control). Moreover, both concentrations of doxycycline caused a statistically significant increase in the value of G2-M/S ratio in A375 cells after 24 h of treatment (1.1 for 200 µM and 1.3 for 400 µM vs. 0.7 in control) and in C32 cells after 48 h of exposure (2.7 for 200 µM and 2.8 for 400 µM vs. 1.5 in control). An increased value of G2-M/S in C32 cells was also observed for a concentration of 200 µM after treatment for 24 h (2.4 vs. 1.4 in control). It should be noted that the incubation of A375 melanoma cells with doxycycline for 48 h did not influence the value of the G2-M/S ratio.

### 2.3. Evaluation of the Viability of A375 and C32 Melanoma Cells after Incubation with Minocycline and Doxycycline

**Figure 3:**
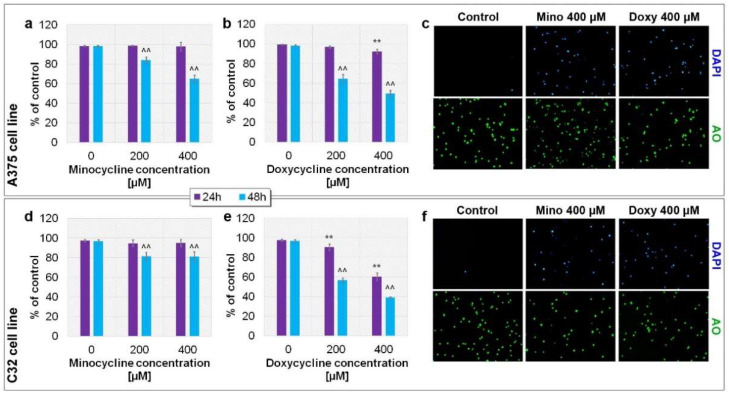
Analysis of melanoma cell viability after the treatment with minocycline and doxycycline. The results are expressed as a percentage of control. Bar graphs present the results for A375 (**a**,**b**) and C32 (**d**,**e**) cell lines. Representative images (**c**,**f**) show analyzed cells stained with acridine orange AO (all cells) and DAPI (dead cells). ** *p* < 0.005 vs. control 24 h; ^^ *p* < 0.005 vs. control 48 h.

The evaluation of cell viability was carried out using imaging cytometry. The analysis indicated that both tested drugs could decrease the viability of amelanotic melanoma cells (Figure 3). Minocycline increased the percentage of dead cells in tested cell lines after only 48 h of incubation. The viability for 200 µM and 400 µM of minocycline after 48 h was 83.8% and 64.9% for A375 cells, and about 81% for C32 cells. In comparison, doxycycline appeared to be more cytotoxic. A significant decrease in cell viability was observed already after 24 h of treatment of C32 cells with 400 µM of doxycycline (60.4% of control). Cell incubation with 200 µM and 400 µM of doxycycline for 48 h resulted in a viability decrease to 64.7% and 49.2% for the A375 cell line and 56.7% and 39.0% for C32 cells, respectively.

Taking into account the obtained results, it was decided that succeeding analyses of the cellular and molecular effects of tested tetracyclines would be made after 48 h of treatment.

### 2.4. Analysis of Reduced Thiols in A375 and C32 Melanoma Cells Treated with Minocycline and Doxycycline

**Figure 4:**
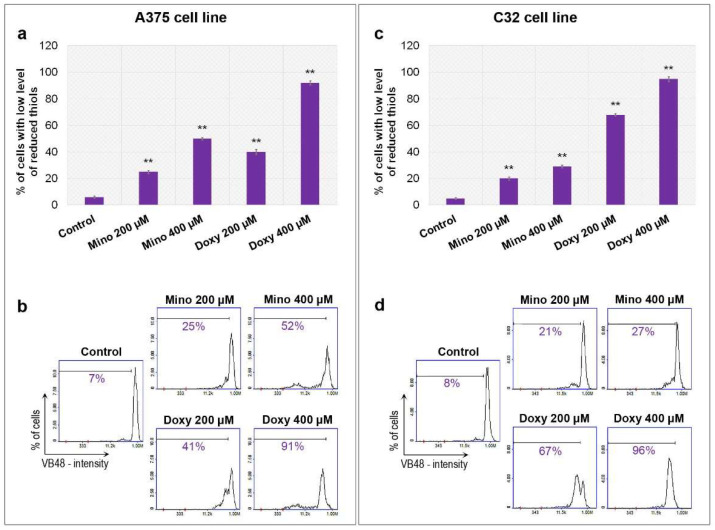
Analysis of intracellular thiols after the treatment of melanoma cells with minocycline and doxycycline. Bar graphs present the results for A375 (**a**) and C32 (**c**) cell lines. Representative histograms (**b**,**d**) display corresponding tested populations. Marked percentages signify cells with a low level of reduced thiols. ** *p* < 0.005 vs. control.

The analysis of intracellular thiols in A375 and C32 melanoma cells was made using an imaging cytometer and the Vitality assay. The cells were stained with VitaBright-48™ which reacted with the reduced form of thiols and then formed a fluorescent product. The examination revealed that minocycline and doxycycline increased the cell population with a low level of reduced thiols (Figure 4). The observed effect was proportional to drug concentration. A decreased level of reduced thiols was observed after the treatment in both cell lines. The influence of doxycycline was significantly stronger than minocycline. The percentage of cells with a low level of reduced thiols after the incubation with 400 µM of doxycycline was 91% and 96% for A375 and C32 cells, respectively. The corresponding results for 400 µM of minocycline were 52% and 27%.

### 2.5. Analysis of Apoptosis of A375 and C32 Amelanotic Melanoma Cells Treated with Minocycline and Doxycycline

**Figure 5:**
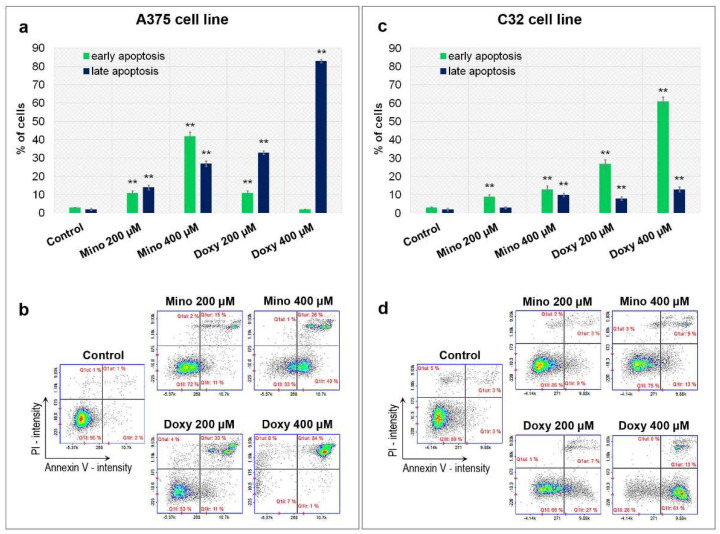
Analysis of apoptosis after the treatment of melanoma cells with minocycline and doxycycline. Bar graphs present the results for A375 (**a**) and C32 (**c**) cell lines. Representative scatter plots (**b**,**d**) display tested populations of stained cells divided by a gate into the subpopulations of non-apoptotic, early, and late apoptotic cells. ** *p* < 0.005 vs. control.

Apoptosis of melanoma cells was examined using image cytometry after 48 h of treatment with minocycline and doxycycline. The annexin V assay was used to label and detect apoptotic cells in the tested population. Moreover, the staining with propidium iodide allowed for differentiation between early and late apoptosis. The analysis revealed that the tested drugs induced apoptosis in both amelanotic melanoma cells (Figure 5). The percentage of apoptotic cells increased proportionally to the drug concentration. It was found that doxycycline appeared to be a stronger inducer of apoptosis than minocycline and A375 melanoma cells were more sensitive to the drug action. It was noted that there was a difference in the rate of induction and apoptosis between the analyzed amelanotic melanoma lines. The largest number of apoptotic cells was observed after incubation with 400 µM of doxycycline and accounted for 85% of the A375 cell population (almost all cells were late apoptotic) and 74% of C32 cells (61% in early apoptosis and 13% in late apoptosis). The corresponding results for 400 µM of minocycline were 66% of A375 cells (40% early apoptosis and 26% late apoptosis) and 22% of C32 cells (13% in early apoptosis and 9% in late apoptosis).

### 2.6. Analysis of Mitochondrial Membrane Potential (ψm) in A375 and C32 Melanoma Cells Treated with Minocycline and Doxycycline

**Figure 6:**
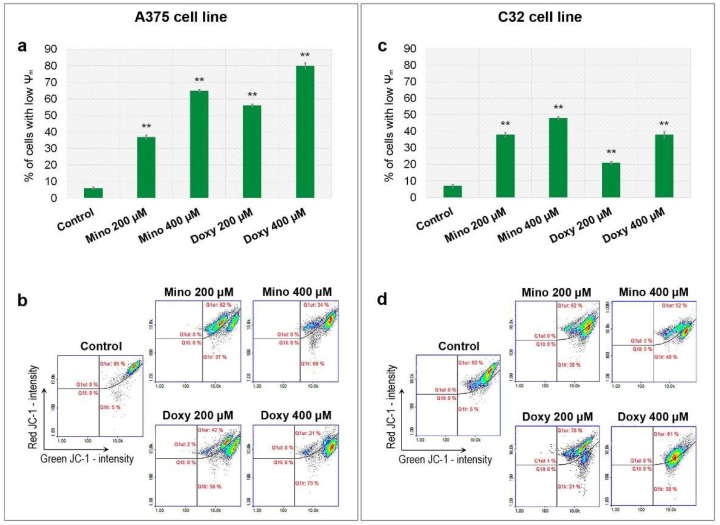
Analysis of mitochondrial membrane potential (ψm) after the treatment of melanoma cells with minocycline and doxycycline. Bar graphs present the results for A375 (**a**) and C32 (**c**) cell lines. Representative scatter plots (**b**,**d**) display tested populations of stained cells divided by a gate into the subpopulations of cells with polarized and depolarized mitochondria. ** *p* < 0.005 vs. control.

The potential of mitochondrial membrane (ψm) was examined cytometrically using JC-1. The stain has the ability to accumulate inside mitochondria in a potential-dependent way. The obtained results indicate that both tested drugs caused a decrease in mitochondrial potential in tested amelanotic melanoma cells (Figure 6). In general, drug-induced mitochondrial depolarization was greater in A375 cells than C32 cells. The largest decrease in ψm was caused by 400 µM of doxycycline in A375 cells. The percentage of cells with depolarized mitochondria in this sample was 79%. An analogous result for minocycline was 66%. On the other hand, the treatment with minocycline had a slightly greater effect on C32 melanoma cells. Minocycline and doxycycline in the highest tested concentration increased the population of C32 cells with depolarized mitochondria to 48% and 38%, respectively.

### 2.7. Analysis of Caspases’ Activity in A375 and C32 Melanoma Cells Treated with Minocycline and Doxycycline

**Figure 7:**
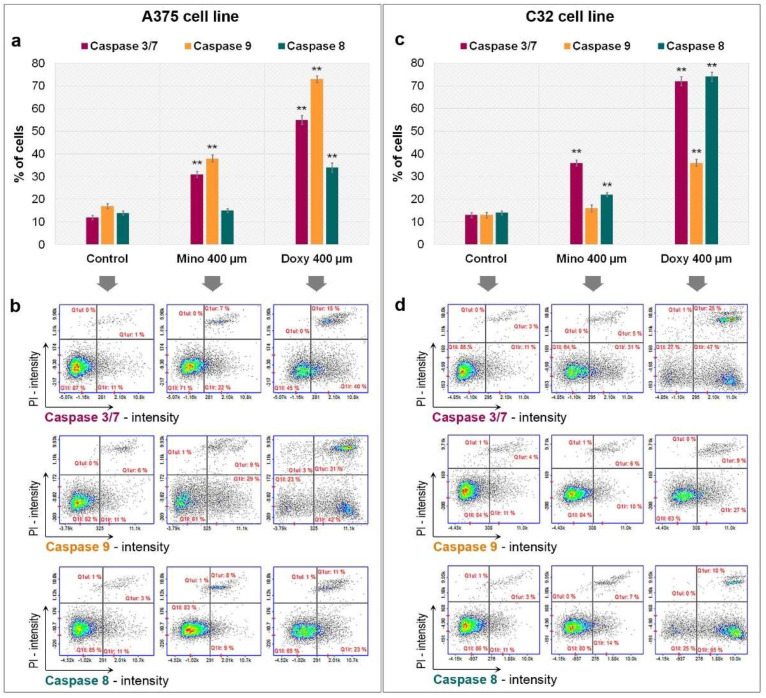
Analysis of caspase activity after the treatment of melanoma cells with minocycline and doxycycline. Bar graphs present the results for A375 (**a**) and C32 (**c**) cell lines. Representative scatter plots (**b**,**d**) present PI- and FLICA-stained cells divided by a gate into the subpopulations of cells without and with activated caspase. ** *p* < 0.005 vs. control.

The study of caspase activity was made using image cytometry. The method is based on fluorochrome-labeled inhibitors of caspases which bind selectively to the enzymes and stain tested cells. The examination revealed that both tested drugs were able to induce the activity of caspases (Figure 7). Doxycycline once again proved to be a stronger drug. The greater effect of doxycycline was related to all tested caspases as well as both cell lines. The percentage of A375 cells with doxycycline-activated caspases 3/7, 9, and 8 was 55%, 73%, and 34%, respectively. Corresponding results for the C32 cell line were 62%, 36%, and 75%. In turn, minocycline activated the enzymes in 29%, 38%, and 17% of A375 cells and 36%, 16%, and 21% of C32 cells, respectively. It is worth noting that the selected cell lines for the study differ in the type of prevailing initiator caspase. Caspase-9 dominated in A375 melanoma cells, while caspase-8 was mainly activated in the C32 cell line.

### 2.8. Analysis of Cell Morphology and Intracellular Level of Cytochrome c in A375 and C32 Melanoma Cells Treated with Minocycline and Doxycycline

**Figure 8:**
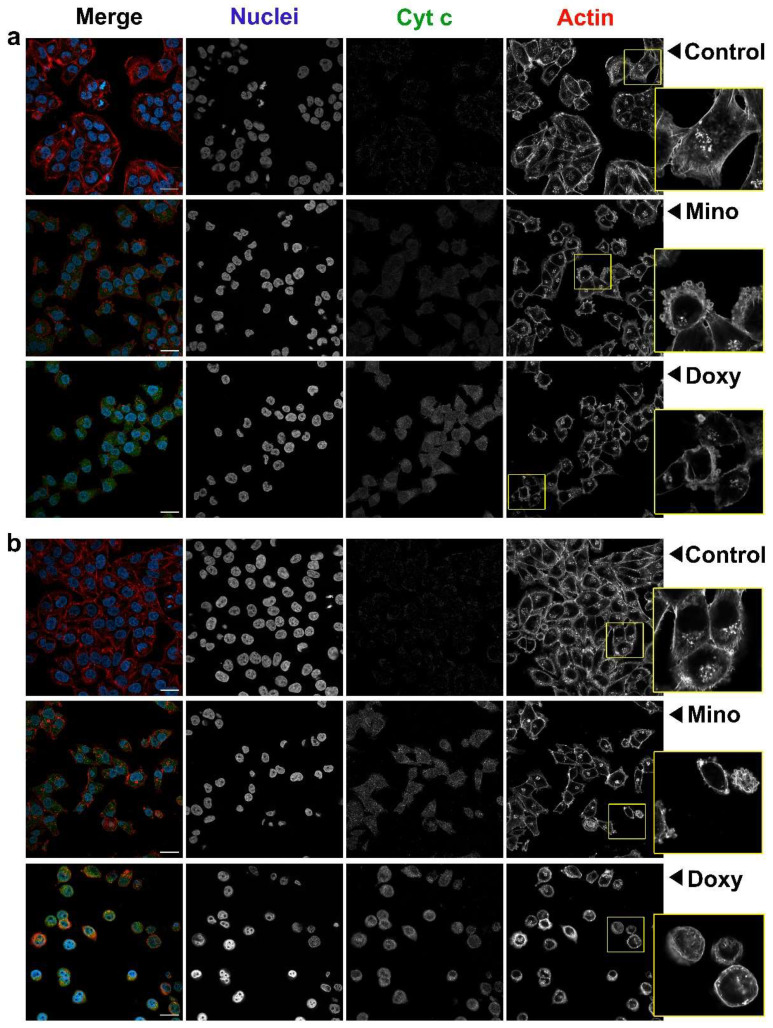
Imaging with confocal microscopy of A375 amelanotic melanoma cells treated with 400 µM minocycline and doxycycline for 24 h (**a**) and 48 h (**b**). Photographs present merged images as well as separate channels for nuclei, cytochrome c, and actin filaments. Scale bar 25 µM.

**Figure 9:**
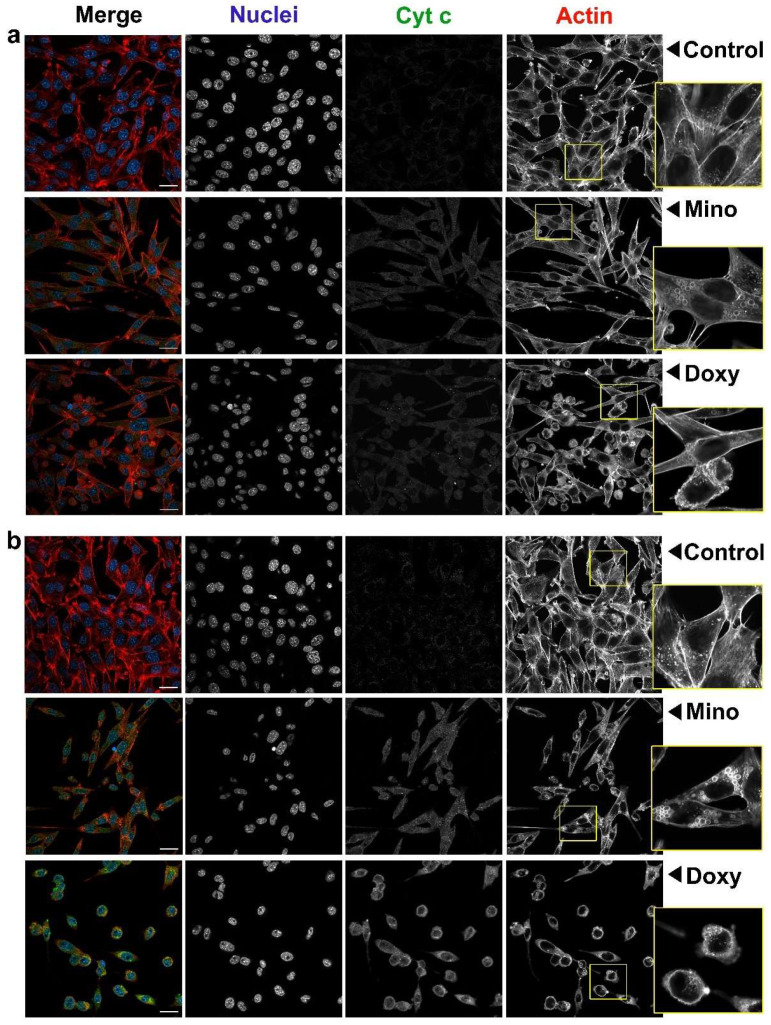
Imaging with confocal microscopy of C32 amelanotic melanoma cells treated with 400 µM minocycline and doxycycline for 24 h (**a**) and 48 h (**b**). Photographs present merged images as well as separate channels for nuclei, cytochrome c, and actin filaments. Scale bar 25 µM.

The anti-melanoma effect of minocycline and doxycycline was also analyzed using confocal microscopy. Cell morphology and the intracellular level of cytochrome c were evaluated. The obtained representative images for A375 and C32 cells are presented in Figure 8 and Figure 9, respectively. The photographs indicate that cells after drug exposure had changed morphology. It was presented that control cells proliferated and tended to adhere tightly to each other. Their shape was similar and characteristic of the cell line. Growing and dividing cells, especially A375 cells, formed a kind of cellular cluster. The treatment caused a growth inhibition which resulted in reduced cell number. Treated melanoma cells grew in few-cell groups or separately. It was mainly doxycycline that caused the loss of intercellular contact and the separate arrangement of cells.

In general, tested tetracyclines reduced cell size and condensed cytoplasm around nuclei. A cellular shape appeared to be less regular and less similar to the control. Cell rounding was observed mainly in A375 cultures and C32 cells after being exposed to doxycycline. The effect was significantly less in C32 cells treated with minocycline. It was noticed that after the treatment nuclei became smaller and irregular with condensed chromatin. In addition, minocycline and doxycycline changed the arrangement of the actin cytoskeleton. The contraction of the actin ring and the appearance of small spherical actin structures around the cell (blebbing) were found in all treated A375 cultures and C32 cells incubated with doxycycline. However, the impairment of the actin skeleton was also triggered by minocycline in C32 cells. In this case, numerous, distinct and round structures made of actin fibers were visible in the cytoplasm.

The confocal microscopic analysis also allowed us to visualize changes in the level of cytochrome c in tested cells. The obtained fluorescence signal of stained cytochrome c in control samples was very weak. The treatment with tetracyclines increased the intensity of fluorescence. The observed effect depended on the incubation time and the drug. The level of cytochrome c in A375 cells significantly rose after 24 h for doxycycline and after 48 h in cells cultured with minocycline and doxycycline. In turn, both examined drugs caused a substantial increase in the level of cytochrome c in C32 melanoma cells after only 48 h of treatment.

### 2.9. Analysis of MITF, ERK1/2, and LC3A/B Level in A375 and C32 Melanoma Cells Treated with Minocycline and Doxycycline

**Figure 10:**
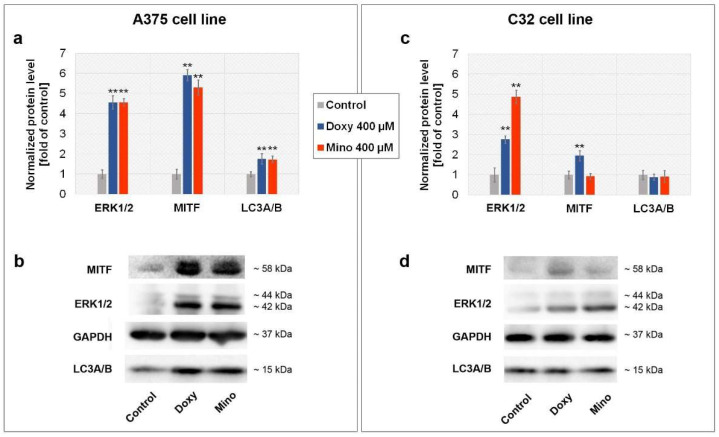
Analysis of ERK1/2, MITF, and LC3A/B level after the treatment of melanoma cells with minocycline and doxycycline. Bar graphs present normalized results for A375 (**a**) and C32 (**c**) cell lines. Corresponding representative blot images (**b**,**d**) for analyzed proteins are also shown. ** *p* < 0.005 vs. control.

The level of selected proteins in tested cells was determined by Western blot analysis. The obtained results are presented in Figure 10 as bar graphs and representative blot images. It was found that both tetracyclines caused a significant increase in ERK1/2 (extracellular signal-regulated kinase 1/2; p44/p42) levels in A375 and C32 cells. The amount of ERK1/2 was around 4.5 times higher in A374 cells for both drugs and C32 cells for minocycline. It was noticed that the effect of doxycycline was weaker in C32 cells. In this case, the increase was found to be around 2.7-fold when compared with the control. Doxycycline and minocycline caused also significant changes in MITF (microphthalmia-associated transcription factor) level in A375 cells. The observed level of the analyzed transcription factor was over 5 times higher in treated A375 cells than in the control samples. The upregulation of MITF in C32 cells was noticed only for doxycycline. The increase, around 2-fold, was significantly weaker than in A375 cells. Doxycycline and minocycline showed no effect on LC3A/B protein level in C32 cells. On the other hand, the drugs increased the amount of LC3A/B by about 70%.

## 3. Discussion

Malignant melanoma is still a serious medical problem. Relatively high mortality, a still-growing number of newly diagnosed cases, and insufficiently effective methods of therapy necessitate melanoma research. One of the uncommon melanoma subtypes is amelanotic melanoma. The estimated incidence of amelanotic melanoma is about 2–8% of melanomas, but the real value can be higher due to misdiagnosis, mainly because of the lack of pigmentation [40]. The amelanotic subtype occurs usually in sun-exposed skin, especially in type I patients in the Fitzpatrick scale, and clinically manifests as red or pink-colored skin, erythematous macule or patch, or as a nodule with or without ulceration [41,42]. Late recognition, delayed treatment, and more aggressive pathological features such as deeper tumor thickness and higher mitotic rate contribute to poor prognosis and short survival [41,43,44].

Previously published studies revealed that minocycline and doxycycline showed anti-cancer potential. The ability of tetracyclines to form complexes with melanin resulted in their accumulation in pigmented tissues [45,46]. Thus, there rose a question about the use of tetracycline accumulation in the therapy of melanoma. Studies on melanotic melanoma cells COLO829 ascertained that minocycline and doxycycline exerted an anti-melanoma effect [38,39]. The tested drugs inhibited cell proliferation, decreased cell viability, and induced apoptosis. The calculated EC_50_ values were 78.6 µM, 31.7 µM, and 13.9 µM for the treatment with minocycline for 24 h, 48 h, and 72 h, respectively. The corresponding results for doxycycline were similar regardless of the time of incubation and accounted for 74.4 µM, 32.3 µM, and 16.3 µM. The presented results show that the treatment of amelanotic melanoma cells required higher concentrations of tested tetracyclines than COLO829. The calculated EC_50_ values for 72 h of treatment with doxycycline and minocycline were 110.4 µM and 234.0 µM for the A375 cell line and 238.9 µM and 273.1 µM for the C32 cell line, respectively. The findings indicate that A375 cells were more sensitive to the anti-melanoma effect of tested tetracyclines than C32 cells, especially for the treatment with doxycycline. Therefore, in the case of tetracyclines, the effectiveness of melanoma therapy may depend on the type of cancer, cell line, and individual drugs. This is a premise for the use of tetracyclines as part of personalized therapy after prior profiling of the patient.

The influence of tetracyclines on normal cells should also be taken into account, considering the potential use of these drugs in melanoma treatment. Previously conducted studies revealed that doxycycline and minocycline inhibited the proliferation of normal epidermal melanocytes. The obtained EC_50_ values for darkly pigmented melanocytes after 24 h of treatment were 40.0 µM and 98.7 µM for doxycycline and minocycline, respectively [47,48]. Lightly pigmented melanocytes were more sensitive to minocycline. In this case, EC_50_ was calculated to be about 48 µM [49]. The observed difference for darkly and lightly pigmented melanocytes suggests that a high content of melanin may protect cells from the tetracycline-induced adverse effect. Simultaneously, it should be noted that although 100 µM of minocycline decreased cell number in tested populations of both melanocyte types, it did not cause an increase in the percentage of dead cells. In turn, studies of doxycycline on fibroblasts demonstrated that the drug had no effect on cell viability after 48 h of treatment at 100 µM and after 72 h of treatment in the concentration of 90 µM [50,51]. Studies of minocycline and doxycycline on human gingival and periodontal fibroblasts showed a significant effect on cell viability only for 48 h of treatment in the concentration range from 300 µM to 1000 µM [52]. On the other hand, LDH cytotoxicity assay indicated that even 7-day-long treatment of human fibroblasts with doxycycline at 104 µM and 416 µM did not decrease cell survival [53]. Moreover, the work on the tetracycline phototoxicity at concentrations up to 200 µM demonstrated that minocycline and doxycycline had no cytotoxic effect on human keratinocytes in the absence of irradiation [54].

Regardless of the in vitro analyses on normal cells, it should be noted that the EC_50_ concentrations of both tested tetracyclines for amelanotic melanoma cells are higher than the therapeutic plasma levels observed during standard antibacterial treatment. The maximum serum concentration for 200 mg of minocycline ranges from 3.0 mg/L (6.6 µM) to 3.36 mg/L (7.3 µM) after oral administration and amounts to 8.75 mg/L (19.1 µM) after intravenous administration. Corresponding results for 200 mg of doxycycline are 2.6 mg/L (5.8 µM)–5.9 mg/L (13.3 µM) after oral administration and 9.3 mg/L (20.9 µM) for the i.v. route. In turn, C_max_ for 500 mg of doxycycline p.o. is 15.3 mg/L (34.4 µM) [55]. The conducted in vitro studies and the general good tolerance of tetracyclines give the potential possibility of using these drugs in higher doses than are currently used in the treatment of infectious diseases. However, the correct selection of tetracycline concentrations to ensure the safety and efficacy of melanoma therapy certainly requires prudence, caution and further research. Consideration should be given to tetracycline accumulation in pigmented tissues, multi-dose therapy, the possibility of local application, and using modern dosage forms.

A distinction between the efficacy of melanotic and amelanotic melanoma cells was found for dacarbazine—a highly cytotoxic alkylating agent and the reference drug for melanoma treatment. The results of the WST-1 assay for melanotic melanoma COLO829 and amelanotic melanoma C32 cells incubated for 24 h with 100 µM of dacarbazine showed a decrease in viable cells by about 46.3% and 27.1%, respectively. Additionally, it was also observed that the influence of dacarbazine on melanotic and amelanotic melanoma cells was similar to the effect on darkly and lightly pigmented normal melanocytes. Cell viability was reduced by approximately 37.1% and 28.8% [56]. In turn, the treatment with dacarbazine at 50 µM for 72 h decreased the viability of melanotic melanoma COLO829 only to 75% [57]. Although analogous conditions for the C32 cell line caused a 55% decrease, the number of dead cells increased by only 6% [58]. In turn, 48 h of treatment of C32 cells with 200 µM and 400 µM of doxycycline caused an increase in the percentage of dead cells to 43.3% and 61.0%, respectively. The observations indicate that dacarbazine, even after 72 h, mainly inhibits cell proliferation but does not induce melanoma cell death. The effect was confirmed by cell cycle analysis and was related to a slight increase in the cell percentage in the G1/G0 phase [58]. In the case of A375 cells, dacarbazine at the near-therapeutic concentration of 40 µM reduced cell viability by less than 15% after 48 h of incubation. Around a 50% decrease in cell survival was noticed at 100 µM [59]. In addition, the study of the BRAF-inhibitor-resistant variant of A375 cells demonstrated a significant reduction in dacarbazine effectiveness. The value of EC_50_ for 72 h of treatment was found to be 288.8 µM [60]. The above data from in vitro studies show some of the limitations and relatively low efficacy of dacarbazine, also observed in clinical practice.

Greater resistance of amelanotic melanoma to the treatment was also noticed for ciprofloxacin. The fluoroquinolone-derivative drug belongs to DNA gyrase inhibitors with anti-tumor potential and, similar to tetracyclines, can form complexes with melanin [61]. Conducted studies on melanoma cells revealed that the drug was more effective against melanotic COLO829 (EC_50_ = 100 µM after 72 h) than amelanotic C32 (EC_50_ = 500 µM after 72 h) [62,63]. Nevertheless, it should be emphasized that the susceptibility of amelanotic and melanotic melanoma cells to the treatment depends finally on the type of drug. A slightly different tendency was found for another fluoroquinolone–moxifloxacin or Mcl-1 inhibitor–MIM1. In the case of these drugs, the 72 h treatment caused a similar effect on COLO829 and C32 cells. The obtained EC_50_ values for moxifloxacin were 150 µM and 110 µM, respectively [64]. In turn, MIM1 decreased cell viability by around 50% in a concentration of 50 µM [57,58].

The significant inhibition of amelanotic melanoma cell proliferation by tested tetracyclines was reflected in the cell cycle profile. The obtained results indicate that minocycline and doxycycline changed the percentage of the cell population in individual phases of the cell cycle. It was found that minocycline, in general, increased the relative ratio of G1/S as well as G2-M/S. The effect was mainly due to a decrease in the number of cells in the S phase. On the other hand, doxycycline decreased the G1/S ratio in A375 cells. The observed increase in the G1/S ratio in C32 cells depended on the drug concentration and incubation time. Moreover, both concentrations of doxycycline increased the value of the G2-M/S ratio only in the C32 cell line. The noticed changes in the cell cycle resulted in the arrest of cell division and a decrease in cell number in populations exposed to tested drugs. The effect was also observed in images from a confocal microscope. The molecular basis of the action could be related to the elevated level of ERK1/2. Extracellular-signal-related kinase 1 (ERK1, p44) and 2 (ERK2, p42) are elements of the mitogen-activated protein kinases (MAPK) pathway. The MAPK pathway is highly conserved in all eukaryotic cells and regulates cell proliferation, survival, differentiation, and apoptosis. In turn, the dysregulation of MAPK signaling is involved in the initiation and progression of cancers. Thus, protein kinases of the pathway, such as ERK1/2, have become a target for cancer therapy [65]. Although inhibitors of MAPKs are usually considered as a potential method of treatment, some drugs stimulate these kinases. Activation of ERK1/2 was noticed for anthracyclines during therapy of neuroblastoma cells, hepatoma cells, cervical carcinoma cells, and breast carcinoma cells [66]. A similar effect was also observed for melanoma cells incubated with cisplatin or phenylethyl resorcinol [67,68]. However, it is worth noting that the effect of ERK1/2 stimulation may cause pleiotropic effects and the interpretation is not unambiguous. On the one hand, activation of ERK signaling may improve cell survival and is thought of as one of the resistance mechanisms, especially regarding targeted therapy with ERK inhibitors [69]. On the other hand, the activation of the pathway can arrest the growth of cells, e.g., by increased expression of molecules such as the cyclin kinase inhibitor protein p21Cip-1/MDA6/WAF1 [70].

Some studies suggest that ERK kinases may activate or up-regulate microphthalmia-associated transcription factor by its direct phosphorylation or by CREB-dependent pathway [71,72]. MITF is a transcription factor specific for melanocytic lineage which regulates many biological processes in melanocytes and melanoma cells. The influence of MITF involves cell differentiation, proliferation, or migration [73]. Previously it was demonstrated that minocycline significantly stimulated the expression of *MITF* and elevated the protein level of the transcription factor in darkly pigmented melanocytes [47]. The observed effect was one of the molecular mechanisms of minocycline-induced skin hyperpigmentation. The presented results indicate a significant increase in MITF level in A375 cells after treatment with minocycline and doxycycline. The increase was observed in C32 cells only for doxycycline. At this stage of research, it is difficult to clearly state the role of MITF in the anti-melanoma action of tested tetracyclines. The analyzed transcription factor is well known for its pro-survival activity, also in melanoma cells [74]. The overexpression of MITF may reduce the efficacy of treatment with BRAF or MEK inhibitors. At the same time, it was found that MITF loss predicted early resistance to targeted therapies [75]. Moreover, the depletion of MITF attenuated the response to induced autophagy, and decreased melanoma immunogenicity and the response to immunotherapy [76,77]. The complexity of the issue is emphasized by the fact that MITF-low melanoma cells are more invasive, whereas high MITF makes melanoma cells more proliferative and differentiated [78]. Thus, the overexpression of ERK1/2 and MITF, observed in most of the tested samples, may play some positive roles; however, the effect requires further research and should be considered regarding the use of tetracyclines as adjuvant therapy for melanoma.

Successive cytometric analysis showed that minocycline and doxycycline decreased cell viability. In general, the effect was proportional to the drug concentration and the incubation time. Doxycycline appeared to be more cytotoxic than minocycline and caused a greater reduction in the number of viable cells. The obtained results for minocycline indicated that A375 cells were slightly more susceptible than C32 cells. In the case of doxycycline, this relationship was the opposite. One of the reasons causing decreased viability was a disturbance of redox homeostasis. The ability of minocycline and doxycycline to induce oxidative stress was previously demonstrated in studies on melanotic melanoma and glioblastoma cells [38,39,79]. Moreover, it was presented that minocycline affected the antioxidant system in lightly pigmented normal melanocytes. The drug elevated the intracellular level of ROS and the percentage of cells with a low level of reduced thiols, and stimulated the activity of the antioxidant enzymes superoxide dismutase and catalase. On the other hand, pretreatment with minocycline significantly attenuated the oxidative stress in melanocytes exposed to the well-known oxidative stress inducers hydrogen peroxide and UVA radiation [49].

An increase in ROS level is characteristic of many anti-cancer drugs, including alkylating agents, anthracycline antibiotics, platinum compounds, mitotic inhibitors, or antimetabolites [80]. Glutathione is the most abundant non-protein thiol acting as an intracellular antioxidant and a regulator of cellular redox state. The reduced form of glutathione (GSH) protects cells from the oxidative damage caused by reactive oxygen or nitrogen species and xenobiotics. In addition, it participates in and can regulate cell signaling, proliferation, differentiation, and death [81,82]. The analysis of the intracellular level of reduced thiols showed that both tested tetracyclines significantly increased cell number with a low level of reduced thiols. It was found that doxycycline was more potent than minocycline. The percentage of cells with a low level of reduced thiols was over 90% for A375 and C32 cells treated with 400 µM of doxycycline. The effect of minocycline was significantly weaker, especially in C32 cells. A general insight into the results led us to conclude that the level of reduced thiols was convergent with the proliferation and viability of investigated melanoma cells.

It is generally believed that the depletion of reduced glutathione (GSH) is one of the common features of apoptotic cells [83]. Apoptosis, a type of programmed cell death, has become the target of cancer therapy over the years. It is a multistep and complex molecular process triggered by intrinsic or extrinsic pathways. The first one is dependent on mitochondria and manifests as the depolarization of mitochondrial membrane, which leads to the release of cytochrome c from the intermembrane space into the cytosol. Released cytochrome c, together with procaspase-9 and Apaf-1, participates in the formation of a multi-protein complex called the apoptosome. The complex activates caspase-9 and, consequently, a cascade of proteolytic effector caspase-3/7. In turn, the extrinsic pathway is triggered by death receptors, mainly tumor necrosis factor (TNF) receptor family members. These receptors transmit extracellular signals via the FAS-associated death domain (FADD) and active caspase-8 that, in turn, causes the activation of the effector caspases. Independently of the activated pathway, apoptosis manifests itself in the form of chromatin condensation, cell shrinkage, membrane blebbing, loss of adhesion, and the externalization of phosphatidylserine [84,85,86].

The presented study indicates that minocycline and doxycycline were able to induce apoptosis in tested amelanotic melanoma cells. The ability to trigger apoptosis was confirmed by the positive results of the annexin V assay, depolarization of the mitochondrial membrane, release of cytochrome c, and activation of caspases. Moreover, some changes characteristic of apoptosis, such as condensed chromatin, reduced cytoplasm, or rearrangement of cytoskeleton, were noticed for treated cells. Over 50% of annexin V-positive cells were found for a population of A375 cells treated with 400 µM of minocycline and doxycycline for 48 h. In the case of C32 melanoma cells, such a high result was only reported for 400 µM of doxycycline. Moreover, it was found that early apoptosis dominated in all treated C32 cells, as well as in A375 cultures incubated with 400 µM of minocycline. In the remaining A375 samples, most of the annexin V-positive cells were in late apoptosis. Imaging of cytochrome c release in A375 cells after 24 h of treatment also confirmed the difference between the drugs. A significant increase in the fluorescence of labeled cytochrome c was observed in these samples only for doxycycline. Apoptosis of amelanotic melanoma cells was also confirmed by caspase analysis. Once again, doxycycline had a stronger effect than minocycline in this regard. Effector caspase-3/7 was activated in all treated cells. However, it is worth noting that the level of initiator caspase-9 was significantly higher than caspase-8 in A375 cells after therapy. In turn, caspase-8 dominated in C32 cells. One of the possible explanations of this phenomenon is the participation of an intracellular catabolic process called autophagy. The process led to the lysosomal degradation of damaged and unused cytoplasmic components in double-membraned vesicles—autophagosomes. Although it is believed that autophagy may promote cell survival, it also contributes to cell death, especially in apoptosis-deficient cancer cells [87,88]. Both apoptosis and autophagy are molecularly cross-regulated and coordinated by intracellular proteins [89]. Several studies showed that initiator caspase-9 positively regulated autophagy and led to autophagosome formation [90,91]. Moreover, it was found that autophagy-competent mitochondrial translation elongation factor TUFM inhibited caspase-8 [92]. So far, the ability to promote autophagy has been demonstrated among others for human monocytic leukemia cells and human glioma cells incubated with minocycline [93,94]. Moreover, protective autophagy was observed in vascular endothelial cells and cardiomyocytes exposed to minocycline, as well as in isolated primary cardiac myocytes cultured with doxycycline [95,96,97]. On the other hand, minocycline inhibited autophagy in human hepatoma cells as well as doxycycline in breast cancer cells [98,99]. Considering all of the above reports, we decided to examine the level of LC3A/B—a marker of autophagy. The study revealed that minocycline and doxycycline upregulated the tested protein only in A375 cells. Thus, the stimulation of LC3A/B was observed only in amelanotic melanoma cells with high levels of caspase-9 activity. In turn, there were no changes in LC3A/B level in C32 cells, dominated by caspase-8. The findings suggest a possible role of autophagy in treatment with tetracyclines; however, a detailed explanation of this issue requires further research.

## 4. Materials and Methods

### 4.1. Chemicals and Reagents

Doxycycline hyclate (C_22_H_24_N_2_O_8_ × HCl × 0.5 H_2_O × 0.5 C_2_H_6_O), minocycline hydrochloride (C23H27N3O7 × HCl). Phalloidin-Atto 565, glycine, Triton X-100 solution, and the antibiotics penicillin and amphotericin B were acquired from Sigma-Aldrich Inc. (Taufkirchen, Germany). Trypsin inhibitor was obtained from Cascade Biologics (Portland, OR, USA). Neomycin was obtained from Amara (Kraków, Poland). Trypsin/EDTA and fetal bovine serum (FBS) were purchased from Cytogen (Zgierz, Poland). Dulbecco’s modified Eagle medium (DMEM), Dulbecco’s phosphate-buffered saline (DPBS), secondary antibody—Alexa Fluor 488 and SYTO Deep Red Nucleic Acid Stain were acquired from Thermo Fisher Scientific (Waltham, MA, USA). Cell Proliferation Reagent WST-1 was produced by Roche GmbH (Mannheim, Germany). Via-1-Cassettes™ (acridine orange and DAPI fluorophores), NC-Slides™ A2, and A8 and the staining solutions Solution 3 (1 μg/mL DAPI, 0.1% triton X-100 in PBS), Solution 5 (400 μg/mL VitaBright-48™, 500 μg/mL propidium iodide, 1.2 μg/mL acridine orange in DMSO), Solution 7 (200 μg/mL JC-1), Solution 8 (1 μg/mL DAPI in PBS), Solution 15 (500 μg/mL Hoechst 33342), and Solution 16 (500 μg/mL propidium iodide) were produced by ChemoMetec (Lillerød, Denmark). Annexin V-CF488A conjugate and Annexin V binding buffer were purchased from Biotium (Fremont, CA, USA). Primary rabbit monoclonal antibodies, anti-GAPDH, anti-LC3A/B, and anti-ERK1/2, were obtained from Cell Signaling (Danvers, MA, USA). Primary mouse monoclonal antibodies, anti-cytochrome c and anti-MITF, were acquired from Santa Cruz Biotechnology Inc. (Dallas, TX, USA). Caspase 9 Assay Kit, Caspase 8 Assay Kit and Caspase 3/7 Assay Kit were purchased from ImmunoChemistry Technologies (Bloomington, MN, USA). The remaining chemicals were acquired from POCH S.A. (Gliwice, Poland) or Sigma-Aldrich (Germany).

### 4.2. Cell Culture and the Treatment

Human skin amelanotic melanoma cell lines A375 and C32 were acquired from ATCC (CRL-1974™, USA). Both cell lines were cultured in Dulbecco’s modified Eagle medium (DMEM) supplemented with inactivated fetal bovine serum to a final concentration of 10%, as well as the antibiotics penicillin (100 U/mL), neomycin (10 μg/mL) and amphotericin B (0.25 μg/mL). Cells were cultured in a 5% CO2 incubator CB 160 (BINDER, Tuttlingen, Germany) at 37 °C with 5% relative humidity. The treatment with doxycycline and minocycline was started 24 h after seeding for melanoma cells. Tested drug solutions were prepared using the culture medium. Cells were detached with trypsin both during cultivation and after treatment.

### 4.3. Screening Analysis of Cells Proliferation

Proliferation of melanoma cells was estimated by the Cell Proliferation Reagent WST-1. The reagent is a tetrazolium salt (slightly red) that can be reduced in viable cells to formazan dye (dark red) by mitochondrial dehydrogenases. WST-1 was added to cells cultured in 96-well microplates in an amount of 10 µL/well 3 h before the measurement. Microplate reader Infinite 200 PRO (TECAN, Männedorf, Switzerland) was used to read absorbance at 440 nm, and 650 nm as a reference wavelength. Control samples were normalized to 100%, and all tested samples were calculated as the percentage of the control.

### 4.4. Cell Cycle Analysis

The cell cycle was analyzed using the NucleoCounter^®^ NC-3000™ fluorescent imaging cytometer (ChemoMetec, Lillerød, Denmark). The test is based on measurements of the DNA content within cells. The evaluation was made after 24 h- and 48 h-long treatments. Melanoma cells in an amount of 1 × 106 were suspended in 0.5 mL PBS and then fixed with 4.5 mL of 70% cold ethanol for at least 12 h at 0–4 °C. After fixation, tested cells were centrifuged. The obtained cell pellets were resuspended in PBS and centrifuged for 5 min at 500× *g*. Then, the cells were stained with Solution 3 according to the producer’s protocol. After the staining, tested cells were loaded into 8-chamber NC-Slides A8™ and analyzed using the Fixed Cell Cycle-DAPI Assay protocol by the NC-3000 image cytometer. The obtained histograms were used to demarcate different phases of cell cycle in the samples. Based on the results, the relative ratios of G1/S and G2-M/S were also calculated.

### 4.5. The Evaluation of Cell Viability

Cell viability was evaluated using a fluorescent imaging cytometer NucleoCounter^®^ NC-3000™ (ChemoMetec, Lillerød, Denmark). The analysis was based on the staining of non-fixed cells with acridine orange (detection of total cells population) and DAPI (detection of dead cells). After the treatment, melanoma cells were detached, centrifuged and resuspended in the growth medium. Then, cell suspension was loaded into Via1-Cassettes™ (ChemoMetec, Lillerød, Denmark) containing the stains and immediately analyzed using the Cell Viability and Cell Count Assays protocol by an NC-3000 image cytometer. The rapid and instant analysis ensures that DAPI penetrates only through a damaged and permeable cell membrane. These conditions mean that only dead cells are DAPI positive.

### 4.6. Confocal Microscopy Imaging

Imaging of A375 and C32 cells was performed using the laser confocal microscope Nikon Eclipse Ti-E A1R-Si and Nikon NIS Elements AR software. The cells were cultured in sterile cover slips in Petri dishes. After the treatment, melanoma cells were fixed with 4% paraformaldehyde and permeabilized with 0.1% Triton X-100. In the next step, the samples were treated with glycine and BSA solutions and then were incubated with primary mouse anti-cytochrome c antibody (1:100) overnight at 4 °C. Afterward, the cells were stained with Alexa Fluor 488 conjugated with the secondary antibody (1:200). SYTO Deep Red Nucleic Acid Stain (1:100) and Phalloidin–Atto 565 (0.6 µM). The dyes allowed us to image cytochrome c, nucleus, and actin filaments, respectively. The prepared cover slips were mounted onto microscopic glass slides in the final step.

### 4.7. Annexin V Assay

The annexin V assay was used to detect cell apoptosis. The principle of the assay is based on the high affinity of annexin V to phosphatidylserine, whose translocation to the outer membrane layer occurs in the early stage of apoptosis. The analysis was performed using a fluorescence image cytometer NucleoCounter^®^ NC-3000™ (ChemoMetec, Lillerød, Denmark) according to the producer’s protocol. After the treatment, 3.0 × 105 cells were suspended in 100 μL of Annexin V binding buffer with 2 µL of Solution 15 (Hoechst33342 stains total population) and 2 µL of FITC-labeled annexin V (Annexin V-CF488A conjugate). Afterwards, tested cells were incubated for 15 min at 37 °C and subsequently centrifuged for 5 min at 400× *g*. The obtained cell pellets were washed twice using Annexin V binding buffer. Finally, the cell pellets were resuspended in 100 µL of Annexin V binding buffer and stained with 2 µL of Solution 16 (propidium iodide stains late apoptotic and necrotic cells). The samples were loaded into 2-chamber NC-Slides A8™ and analyzed immediately using the Annexin V Assay protocol. The obtained scatterplots were used to demarcate the percentage of non-apoptotic cells, as well as early and late apoptotic cells.

### 4.8. The Estimation of Intracellular Thiol Level

Intracellular thiol level was estimated using the fluorescence imaging cytometer NucleoCounter^®^ NC-3000™ (ChemoMetec, Lillerød, Denmark). The crucial step of the assay is cell staining with Solution 5 (VitaBright-48™). The reagent allows the marking of cells with a high level of reduced thiols, such as GSH. After the treatment, melanoma cells were suspended in PBS (2 × 106 cells/mL) and stained with VitaBright-48™ (10 µL was added into 190 µL of the cell suspension). Next, the samples were loaded into 8-chamber NC-Slides A8™ and measured using the Vitality Assay protocol in the NC-3000 image cytometer. The obtained histograms were used to demarcate cell subpopulations with high and low levels of reduced thiols.

### 4.9. Mitochondrial Potential Analysis

The analysis of mitochondrial transmembrane potential (ΔΨm) was made by the use of the fluorescence image cytometer NucleoCounter^®^ NC-3000™ (ChemoMetec, Lillerød, Denmark). The principle of the assay is related to the accumulation of a fluorescent cationic dye JC-1 in the mitochondria in a potential-dependent manner. The dye accumulates inside the mitochondria with the high transmembrane potential (healthy cells). A high concentration leads to the aggregation of JC-1 (red fluorescent). In apoptotic cells (mitochondria with low transmembrane potential) JC-1 is localized in the cytoplasm (green fluorescence). After the treatment, 1.0 × 10^6^ cells were suspended in 12.5 μL of Solution 7 (JC-1) and then incubated at 37 °C for 10 min. Next, the cells were centrifuged for 5 min at 400× *g* and washed twice with PBS. The obtained cell pellets were resuspended in 0.25 mL of Solution 8 (DAPI). Finally. the samples were loaded into 8-chamber NC-Slides A8™ and analyzed immediately using the Mitochondrial Potential Assay protocol. The results in the form of scatterplots were used to demarcate the percentage of cells with a polarized or depolarized mitochondrial membrane.

### 4.10. Caspase Activity Assay

The caspase activity was analyzed by the use of fluorochrome-labeled inhibitor of caspases (FLICA reagent). The inhibitors bind selectively to the form of active caspase. In turn, the unbound inhibitors diffuse out of the cells and are washed away during sample preparation. After the treatment, melanoma cells were suspended (4 × 106 cells/mL) and stained with FLICA reagent and Hoechst 33342 (5 µL and 2 µL/93 µL the cell suspension, respectively). Next, the samples were incubated at 37 °C for 60 min and then washed twice with 400 µL of apoptosis wash buffer. Finally, the cells were resuspended in 100 μL apoptosis wash buffer and stained with propidium iodide (10 μg/mL). The obtained samples were loaded into 2-chamber NC-Slides A2™ and analyzed immediately in the fluorescence image cytometer NucleoCounter^®^ NC-3000™ (ChemoMetec, Lillerød, Denmark) using the Caspase Assay protocol. The results in the form of scatterplots were used to demarcate the percentage of cells without and with active caspases (FLICA positive).

### 4.11. The Analysis of Protein Concentration

The concentration of total protein in cell lysates was determined using Pierce™ BCA Protein Assay Kit (Thermo Fisher Scientific Inc., Waltham, MA, USA), according to the producer’s instruction. The assay is based on the ability of protein to reduce Cu^2+^ to Cu^1+^ in an alkaline medium, as well as the selective and sensitive colorimetric detection of Cu^1+^ by bicinchoninic acid (BCA). The measurement of absorbance was performed at 562 nm using a microvolume spectrophotometer DS-11 (DeNovix, Wilmington, DE, USA).

### 4.12. Western Blotting Analysis

After the treatment, melanoma cells were lysed using RIPA buffer containing phosphatase and protease inhibitors. Prepared lysates were centrifuged at 12,000 rpm for 10 min at 4 °C and stored at −86 °C until assessment of total protein concentration and Western blotting analysis. Protein extracts (20 µg/lane) were separated on an 10% SDS–polyacrylamide gel electrophoresis and transferred to PVDF membranes (Sigma-Aldrich Inc., Taufkirchen, Germany). The membranes were incubated for 1 h in blocking buffer (a solution of 5% non-fat milk and TBST (Tris-buffered saline with Tween 20) and washed with TBST. The analyzed proteins were detected using the primary monoclonal antibodies rabbit anti-GAPDH (1:1000), rabbit anti-LC3A/B (1:1000), mouse anti-MITF (1:1000), and rabbit anti-ERK1/2 (1:1000), diluted in blocking buffer. The incubation was carried out overnight at 4 °C. Next, the membranes were washed with TBST and incubated for 1.5 h at room temperature with appropriate horseradish-peroxidase-conjugated secondary antibody, diluted previously in blocking buffer (1:10,000). In the final step, the protein signals were detected using ECL reagent. The analysis was made using G:Box Chemi-XT4 Imaging System and GeneTools Software (Syngene, Cambridge, UK). The obtained results were normalized using the level of GAPDH and expressed as the percentage of control.

### 4.13. Statistical Analysis

Statistical analysis of the results was performed using GraphPad Prism 6.01 soft-ware. Mean values of at least three separate experiments performed in triplicate (*n*= 9) ± standard deviation of the mean (SD) were calculated. The results were analyzed statistically by one-way ANOVA and two-way ANOVA, as well as Dunnett’s and Tukey’s multiple comparison tests. The Kolmogorov–Smirnov test checked the compliance of the distribution results and the Brown–Forsythe test checked that the variances of the compared groups met the homogeneity assumption. In all cases, statistical significance was found with a *p*-value lower than 0.05.

## 5. Conclusions

In summary, the obtained results indicated the anti-cancer potential of minocycline and doxycycline against amelanotic melanoma cells. Considering this action, doxycycline appeared to be a more potent drug than minocycline. Nevertheless, both tetracyclines inhibited cell proliferation, decreased cell viability, and induced apoptosis. The findings were confirmed by the analysis of cell cycle, reduced thiols, annexin V and mitochondrial membrane potential, as well as confocal microscope images. A375 and C32 cells differed in their sensitivity to the treatment and the type of dominant initiator caspase. The study revealed that the action of minocycline and doxycycline might be related to autophagy induction in A375 cells. This finding, as well as the observed stimulation of ERK1/2 and MITF, requires further study. It seems that the effects may be of great importance for using tetracyclines in the potential adjuvant therapy of amelanotic melanomas. We believe that the presented results will contribute to better understanding tetracyclines’ pharmacology and their molecular mechanisms of anti-melanoma action.

## Figures and Tables

**Table 1:** The calculated EC_50_ values for minocycline and doxycycline after 24 h, 48 h and 72 h treatment of A375 and C32 melanoma cells.

*Cell Line*	A375			C32		
Incubation time	24 h	48 h	72 h	24 h	48 h	72 h
Minocycline (µM)	528.4	307.8	234.0	1040.0	651.9	273.1
Doxycycline (µM)	518.5	226.1	110.4	1270.0	416.0	238.9

## Data Availability

The data presented in this study are available upon request from the corresponding author.
